# Characterization of basal and lipopolysaccharide-induced microRNA expression in equine peripheral blood mononuclear cells using Next-Generation Sequencing

**DOI:** 10.1371/journal.pone.0177664

**Published:** 2017-05-26

**Authors:** Nicholas J. Parkinson, Virginia A. Buechner-Maxwell, Sharon G. Witonsky, R. Scott Pleasant, Stephen R. Werre, S. Ansar Ahmed

**Affiliations:** 1 Department of Large Animal Clinical Sciences, Virginia-Maryland College of Veterinary Medicine, Virginia Polytechnic and State University, Blacksburg, Virginia, United States of America; 2 Laboratory for Study Design and Statistical Analysis, Virginia-Maryland College of Veterinary Medicine, Virginia Polytechnic and State University, Blacksburg, Virginia, United States of America; 3 Department of Biomedical Sciences and Pathobiology, Virginia-Maryland College of Veterinary Medicine, Virginia Polytechnic and State University, Blacksburg, Virginia, United States of America; Cornell University, UNITED STATES

## Abstract

The innate immune response to lipopolysaccharide contributes substantially to the morbidity and mortality of gram-negative sepsis. Horses and humans share an exquisite sensitivity to lipopolysaccharide and thus the horse may provide valuable comparative insights into this aspect of the inflammatory response. MicroRNAs, small non-coding RNA molecules acting as post-transcriptional regulators of gene expression, have key roles in toll-like receptor signaling regulation but have not been studied in this context in horses. The central hypothesis of this study was that lipopolysaccharide induces differential microRNA expression in equine peripheral blood mononuclear cells in a manner comparable to humans. Illumina Next Generation Sequencing was used to characterize the basal microRNA transcriptome in isolated peripheral blood mononuclear cells from healthy adult horses, and to evaluate LPS-induced changes in microRNA expression in cells cultured for up to four hours. Selected expression changes were validated using quantitative reverse-transcriptase PCR. Only miR-155 was significantly upregulated by LPS, changing in parallel with supernatant tumor necrosis factor-α concentration. Eight additional microRNAs, including miR-146a and miR-146b, showed significant expression change with time in culture without a clear LPS effect. Target predictions indicated a number of potential immunity-associated targets for miR-155 in the horse, including SOCS1, TAB2 and elements of the PI3K signaling pathway, suggesting that it is likely to influence the acute inflammatory response to LPS. Gene alignment showed extensive conservation of the miR-155 precursor gene and associated promoter regions between horses and humans. The basal and LPS-stimulated microRNA expression pattern characterized here were similar to those described in human leukocytes. As well as providing a resource for further research into the roles of microRNAs in immune responses in horses, this will facilitate inter-species comparative study of the role of microRNAs in the inflammatory cascade during endotoxemia and sepsis.

## Introduction

Excessive activation of the innate immune system can be as damaging to the host as the insult that triggered it. The systemic inflammatory response to bacterial lipopolysaccharide (LPS), mediated via toll-like receptor 4 (TLR4) is a case in point, and forms a critical element of the morbidity and mortality of gram-negative sepsis. Improved understanding of the regulation of this system could facilitate the design of therapeutic interventions that could limit the damaging consequences of the inflammatory response without inducing a deleterious state of immunosuppression, potentially improving outcomes for intensive care patients.

One aspect of TLR signaling regulation that is gaining increasing attention is the role of post-transcriptional regulation by microRNAs (miRNAs) [1]. These are a recently discovered class of non-coding RNA, 17–25 nucleotides in length, that function via interactions with messenger RNA (mRNA), predominantly acting to suppress protein production. Targeting of mRNAs requires near exact complementarity with a six to eight nucleotide seed sequence at the 5’ end of the miRNA, which, due its short length, will have possible matches in a large number of mRNA sequences. Target sites for multiple miRNAs can also be present in a single mRNA [2, 3]. A number of miRNAs have been shown to regulate elements of the TLR4 signaling cascade. For example, miR-146a suppresses a number of intermediate signaling proteins, resulting in reduced activity of the transcription factor nuclear factor-κB (NFκB) and reduced production of inflammatory cytokines [4]. Other miRNAs target cytokines directly. MicroRNA-16, miR-125b and miR-187 all reduce TNFα expression by reducing mRNA stability, while miR-181a targets IL-1α mRNA in a similar manner [5–8].

The horse is a potential model for the study of the systemic response to LPS in naturally-occurring disease. In some ways, the equine response is a closer reflection of human disease than commonly used rodent models. Humans and horses are both exquisitely sensitive to LPS, whereas rodents can tolerate doses several orders of magnitude higher [9–12]. Furthermore, LPS-associated systemic inflammatory responses (referred to clinically as ‘endotoxemia’) are readily observed in common equine diseases [13]. These include septic conditions such as neonatal sepsis, which shares many similarities to human Gram-negative sepsis, but also non-septic gastrointestinal diseases, in which lipopolysaccharide is translocated across damaged intestinal mucosa. While some elements of TLR4 signaling are well characterized in the horse, miRNA responses are as yet unexplored. Indeed, until recently, bioinformatic predictions [14] of miRNAs in the equine genome had remained largely unvalidated. Increasing availability of Next Generation Sequencing technology has brought rapid advances in the field, and miRNA expression has now been characterized in a number of equine tissues, including liver, colon, muscle and plasma [15, 16]. There is however no data on expression in equine inflammatory cells or the role of miRNAs in equine inflammatory responses, hindering any cross-species comparisons.

We hypothesized that exposure to LPS would induce differential expression of miRNAs in equine peripheral blood mononuclear cells (PBMCs), and that the miRNA response would be comparable to that observed in humans. This study combined Next-Generation Sequencing (NGS) technology with a simple *in vitro* model of LPS exposure to assess transcriptome-wide changes in miRNA expression, in addition to providing a first description of basal expression in equine peripheral blood mononuclear cells. This identified one principal LPS-responsive miRNA, shared with other species including humans.

## Materials and methods

### Animals

Eight healthy adult geldings were selected from the University riding herd. These consisted of four Warmbloods, two Thoroughbreds, one Thoroughbred cross and one Tennessee Walking Horse, aged nine to twelve years. All animals had been free from illness or injury within the preceding twelve months, and were confirmed healthy by clinical examination and complete blood count. None had received any vaccinations or other medications for at least one week. Animal procedures were approved by the Virginia Tech Institutional Animal Care and Use Committee, protocol number 14–244. Details of horses and sample numbers used in each stage of the experiment are provided in the supplementary information (S1 File).

### Cell isolation, culture and stimulation

One hundred mL blood was collected aseptically from each animal into acid citrate dextrose tubes, and processed within two hours. Peripheral blood mononuclear cells were isolated by gradient density centrifugation over 59% Percoll® (Sigma-Aldrich, St. Louis, MO) gradients and re-suspended in RPMI-1640 medium with 25 mmol/L HEPES buffer (Gibco®, Thermo Fisher Scientific, Waltham, MA), 10% low-endotoxin heat-inactivated fetal calf serum (Gibco®, Thermo Fisher Scientific), 2 mmol/L L-glutamine (Gibco®, Thermo Fisher Scientific), 1 mmol/L sodium pyruvate (Sigma-Aldrich, St. Louis, MO), 50 u/mL penicillin and 50 μg/mL streptomycin (Gibco®, Thermo Fisher Scientific), to a concentration of 2 x 10^6^ cells per mL. The harvested cell population, evaluated on a Cytospin™ (Thermo Fisher Scientific) preparation, consisted of a median of 77% lymphocytes (range 67–86%), 21% monocytes (range 14–27%), 0.5% neutrophils (range 0–5%), and 0.5% basophils (range 0–1.7%). Cell viability, assessed by Trypan blue exclusion, was ≥95%. Baseline samples were collected prior to culture, and the remaining aliquots of PBMCs from each horse were cultured on a 24-well plate at 37°C and 5% CO2 in the above medium, with the addition of either *E*. *coli* LPS O111:B4 (BioXtra; Sigma-Aldrich, St. Louis, MO) at 10ng/mL or culture medium alone. Cells were cultured for two, four and eight hours. The supernatant was removed after centrifugation and stored at -80°C for later cytokine analysis. Cells were immediately lysed on the plate using a phenol / guanidine thiocyanate lysis buffer (QIAzol® Lysis Reagent; Qiagen, Valencia, CA) and the lysates stored at -80°C pending RNA extraction.

### Cytokine analysis

To confirm effective cell stimulation, the key pro-inflammatory cytokines TNFα, IL-4, IL-17, interferon-α (IFNα) and interferon-γ (IFNγ) and the anti-inflammatory cytokine IL-10 were measured in the culture supernatants from both LPS-stimulated and unstimulated PBMCs from each horse (*n* = 8). Measurements were taken at two, four and eight hours. Tumor necrosis factor alpha was measured using a commercially available equine-specific antigen-capture sandwich enzyme-linked immunosorbent assay kit (Genorise Scientific Inc., Glen Mills, PA) according to the manufacturer’s instructions. Interleukin-4, IL-10, IL-17, IFNα and IFNγ were measured using a multiplex fluorescent bead-linked immunoassay [17] by a commercial laboratory (Horse Cytokine 5-plex Assay, Cornell Veterinary Diagnostics, Ithaca, NY).

### RNA extraction

Total RNA was extracted from cell lysates using miRNeasy spin columns (Qiagen, Valencia, CA) according to the manufacturer’s instructions, with on-column DNAse treatment to eliminate residual genomic DNA. Spectrophometric assessment (Nanodrop; Thermo Scientific, Waltham, MA) confirmed that RNA purity was adequate (260/280 ratio 2.0–2.1 in all samples). The 260/230 ratio was between 1.8 and 2.2 for 80% of samples, although lower values (0.6–1.7) were obtained in 11 samples, likely reflecting minor contamination with guanidine thiocyanate from the extraction procedure. As this measure has little correlation with efficiency of downstream analyses [18], these samples were not excluded, and did not have appreciable differences in sequence read count or quality, or PCR cycle threshold values. The RNA integrity in samples used for sequencing was confirmed to be sufficient by electrophoresis (Bioanalyzer 2100; Agilent, Santa Clara, CA), with an RNA Integrity Number between 8.4 and 9.3 in all samples. The samples were diluted to 220 ng/μL for sequencing, and to 66 ng/μL for PCR, based on fluorometric quantification (Qubit™, Thermo Fisher Scientific).

### Next-Generation Sequencing for miRNA expression

Short-read Illumina Next Generation Sequencing (NGS) was used to measure the complete PBMC miRNA transcriptome in an initial sample of four horses. Basal expression was determined in unstimulated cells prior to culture, and samples from LPS-stimulated and unstimulated cells at two and four hours were used to determine differential expression.

Small RNA library construction was performed using a TruSeq Small RNA preparation kit (Illumina, San Diego, CA). Adaptors were ligated to the 5’-phosphate and 3’-hydroxyl ends of small RNAs from 1μg total RNA. The ligated small RNAs were reverse transcribed and ‘barcoded’ with a unique identifying sequence via PCR amplification for 11 cycles. The 147 bp (miRNA) and 157 bp fractions were extracted from pooled samples using Pippin Prep (Sage Biosciences, Beverly, MA). Pools were cleaned using Agencourt AmpureXP magnetic beads (Beckman Coulter, Indianapolis, IN), quantitated using a Quanti-iT dsDNA HS Kit (Invitrogen, Waltham, MA), and sizes validated on an Agilent 2100 Bioanalyzer. Libraries were clustered onto a flow cell using a TruSeq SR Cluster Kit v3 (Illumina). Sequencing was performed over 50 cycles on a HiSeq 2500 sequencer.

Adapter sequences, excessively short reads and low quality reads were trimmed from the raw sequence data using Btrim [19]. The trimmed reads were aligned to the equine genome assembly Equ Cab 2.0 using Bowtie2 [20] in end-to-end mode, with a seed length of 14 nucleotides, allowing no mismatches. Reads mapping to multiple genomic locations were assigned to the locus with the best alignment score, or if alignment scores were equal at different loci the read was assigned to one using a pseudo-random number generator. Aligned reads corresponding to known equine miRNAs were identified by reference to the miRNA database miRBase, Release 21 [21], and counted using the Python script HTSeq Count [22]. Expression data were normalized across by dividing the expression values for each library by a scaling factor, calculated as the median for each library of the ratios of the individual read counts to the geometric mean count for each miRNA [23].

### Quantitative reverse transcription PCR (qRT-PCR)

Selected expression changes were validated using qRT-PCR in a larger sample set to increase statistical power. This sample set consisted of the samples used for sequencing and cells treated in the same manner from an additional four horses (total *n* = 8). The time course of the response was investigated further by including the eight hour time point from each horse in this analysis. Identity of selected sequences (Table 1) to human miRNAs was confirmed by BLASTN analysis.

**Table 1:** Primers used for miRNA qRT-PCR.

miRNA	Catalog no.	Mature sequence	miRBase accession no. (equine)	miRBase accession no. (human)
eca-miR-155-5p	477927_mir	UUAAUGCUAAUCGUGAUAGGGGU	MIMAT0013182	MIMAT0000646
eca-miR-146a-5p	478399_mir	UGAGAACUGAAUUCCAUGGGUU	MIMAT0013065	MIMAT0000449
eca-miR-146b-5p	478513_mir	UGAGAACUGAAUUCCAUAGGCU	MIMAT0012891	MIMAT0002809
Control: eca-miR-26a-5p	477995_mir	UUCAAGUAAUCCAGGAUAGGCU	MIMAT0012975	MIMAT0000082
Control: eca-miR-103	478253_mir	AGCAGCAUUGUACAGGGCUAUGA	MIMAT0013105	MIMAT0000101

Quantitative reverse transcription PCR was performed using Taqman® Advanced miRNA assays (Applied Biosystems, Carlsbad, CA) according to the manufacturer’s instructions. All assays were performed in triplicate. Normfinder [24] was used to select candidate endogenous controls from NGS expression data.

### Statistical analysis

Differential expression was evaluated from the NGS data by mixed linear models in the Bioconductor package limma for R (Version 3.2.3; The R Foundation for Statistical Computing, Vienna, Austria), with moderation of the standard errors of estimated expression changes by an empirical Bayes method [25]. ‘Horse’ was assigned as a random effect to account for repeated measures. Sequences with very low expression were filtered out prior to analysis, retaining only those miRNAs with a normalized read count greater than 2.0 in at least four samples (234 miRNAs). *Post hoc* contrasts were used to determine the effects of LPS treatment and time. The Benjamini-Hochberg procedure was used to control the false discovery rate (< 0.05).

Two-way hierarchical clustering was performed on transcriptome-wide expression data from baseline and cultured samples using mixOmics for R [26]. Data with a standard deviation less than 1.0 were filtered out prior to analysis, leaving a total of 278 miRNA sequences. The Pearson correlation coefficient was used as a distance metric, and the average linkage method was employed [27].

Quantitative reverse transcription PCR data were analyzed in SPSS Version 22 (IBM Corporation, Armonk, NY) using mixed linear models, accounting for repeated measures within each animal. Normalized ΔCT data were used for analysis. The analysis used was tolerant of missing data points that occurred at individual time points due to technical failure in three horses (see S1 File), and from exclusion of one extreme outlying data point (in which the endogenous control CT deviated from the mean by over five standard deviations). *Post hoc* tests were performed using the Bonferroni correction to account for multiple comparisons. Cytokine data were analyzed in the same manner, after square-root transformation to stabilize variance and fulfil the model assumptions. Pearson correlation coefficients were calculated for pairwise correlations between miRNA -ΔΔCT values and square-root transformed supernatant cytokine concentrations, across all time points. Correlation *p*-values were adjusted for multiple comparisons using the Bonferroni method. Significance for all tests was set at *p* < 0.05.

### Target prediction and functional annotation

Predictions of miRNA targets in the equine genome were performed in TargetScan release 6.2 (www.targetscan.org/vert_61). Only genes containing conserved target sites were considered. A threshold of total context+ score [28] < -0.1 was set. Further target predictions were made by using the complementary prediction algorithm RNA22 version 2 (https://cm.jefferson.edu/rna22) [29] to interrogate a list of candidate targets associated with inflammation and immunity. These candidates were extracted from miRTarBase, a database of experimentally validated targets in other species [30]. The following parameters were used in RNA22: 7-nucleotide seed size with maximum 1 unpaired base; minimum 12 paired-up bases in heteroduplex; maximum folding energy of heteroduplex -12 Kcal/mol; maximum 1 G:U wobble in seed region; sensitivity 87%, specificity 25%. Lists of human gene orthologs from the TargetScan output were imported into PANTHER version 11 [31] (www.pantherdb.org) for functional annotation using gene ontology (GO) terms for pathways and biological processes (Gene Ontology Database release 2016-08-22). Statistical over-representation of gene ontology terms was assessed with a binomial test against reference sets of equine genes, using Bonferroni’s correction as a conservative correction for multiple comparisons.

### miRNA gene conservation analysis and transcription factor binding site prediction

The encoding gene and flanking sequences for the equine miR-155 precursor were aligned to the human and murine genomes to assess conservation between species. Genetic sequences and annotations were obtained from Ensembl (www.ensembl.org), using human genome assembly GRCh38.p5 (GCA_000001405.20), equine genome assembly Equ Cab 2.0 (GCA_000002305.1) and murine genome assembly GRCm38.p4 (GCA_000001635.6) for comparison. Sequences of the region of interest in each species were imported in FASTA format into mVISTA [32] (http://genome.lbl.gov/vista) for alignment, using a reference human sequence extending from the promoter region 1500bp upstream of the first exon, to the end of the last exon. Cross-species conservation was analyzed using rankVISTA. Putative transcription factor binding sites were identified with rVISTA, using TRANSFAC matrices for NFKB and AP-1 (associated with TLR4 signaling). Prediction parameters were set to minimize the sum of type I and type II errors.

## Results

### Characterization of the microRNA transcriptome in equine peripheral blood mononuclear cells

High throughput sequencing of the initial sample set of PBMCs from four horses generated a mean of 4.3 x 10^6^ reads per sample after quality controls. Reads had a mean length of 21 nucleotides, with a mean Phred score of 38. Ninety-three percent of reads mapped to the equine genome, and of these, 58% corresponded to miRNA precursor genes annotated in miRBase Version 21, and 57% corresponded to known mature miRNAs. A total of 372 mature miRNAs were detected, of 690 previously described in the equine genome. Of these, 294 were detected in at least one sample in every horse, and 226 were expressed in all samples. The ten most abundant miRNAs in baseline samples (Table 2) based on normalized read count accounted for 71% of the total mapped miRNA reads. Full details of all miRNAs detected, including genomic location, are given in the supplementary data (S2 File).

**Table 2:** The ten most abundant miRNAs in unstimulated peripheral blood mononuclear cells (time 0).

miRNA	Mean normalized count	mirBase accession number
eca-miR-21	688,668	MIMAT0013029
eca-let-7g	176,440	MIMAT0013075
eca-miR-26a	155,623	MIMAT0012975
eca-miR-150	115,270	MIMAT0013011
eca-miR-148a	86,931	MIMAT0012935
eca-let-7f	79,825	MIMAT0013111
eca-miR-142-5p	66,547	MIMAT0013022
eca-miR-191a	50,440	MIMAT0013079
eca-miR-27a	46,283	MIMAT0012988
eca-miR-30d	35,439	MIMAT0013006

### Differential expression of miRNAs in response to LPS

#### Next Generation Sequencing data

**Fig 1:**
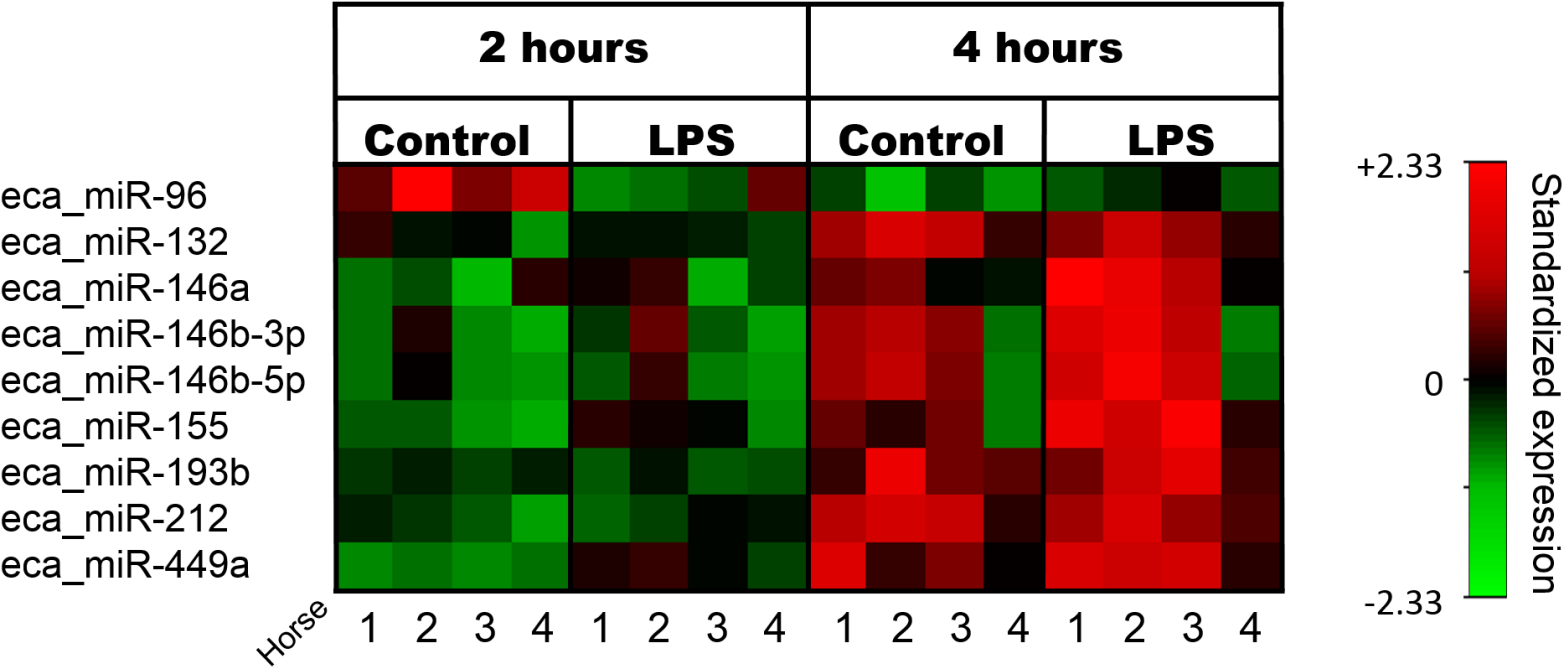
NGS analysis of differential miRNA expression in cultured equine PBMCs. Heatmap of significant expression changes in LPS-stimulated and unstimulated cells after 2 and 4 hours in culture. Expression data have been standardized as *z*-scores for each miRNA. For details, see Table 3.

After correction for multiple comparisons, only miR-155 showed a statistically significant difference in expression between LPS-treated cells and controls (*q*-value 0.036 at 4 hours). Nine miRNAs showed statistically significant expression changes with time (Fig 1 and Table 3). miR-146a, miR-449a and miR-96 showed a tendency towards changes in expression with LPS, but these did not retain statistical significance after the Benjamini-Hochberg procedure.

**Table 3:** Details of miRNA expression changes in cultured equine PBMCs.

		Treatment Contrasts						Time Contrasts					
		LPS vs Ctrl, 2h			LPS vs Ctrl, 4h			4h vs 2h, Ctrl			4h vs 2h, LPS		
miRNA	Mean Count	F.C.	*P*	*Q*	F.C.	*P*	*Q*	F.C.	*P*	*Q*	F.C.	*P*	*Q*
**miR-96**	9.2 ± 4.0	0.5	0.0022	0.29	1.3	0.17	>0.99	0.4	**0.0002**	0.011*	1.0	0.80	>0.99
**miR-132**	1000 ± 670	1.0	0.88	0.97	0.9	0.66	>0.99	1.9	**<0.0001**	0.007*	1.8	**0.0003**	0.019*
**miR-146a**	28000 ± 6800	1.1	0.79	0.95	1.2	0.013	>0.99	1.2	0.088	0.65	1.4	**0.0004**	0.019*
**miR-146b-3p**	150 ± 130	1.4	0.24	0.97	1.3	0.39	>0.99	2.8	**0.0011**	0.042*	2.5	0.0023	0.077
**miR-146b-5p**	7300 ± 5500	1.2	0.76	0.93	1.3	0.23	>0.99	2.6	**0.0014**	0.047*	2.9	**0.0002**	0.016*
**miR-155**	9200 ± 4000	1.4	0.0081	0.44	1.5	**0.0001**	0.036*	1.6	**0.0007**	0.030*	1.8	**<0.0001**	0.0016*
**miR-193b**	180 ± 110	0.9	0.23	0.93	1.1	0.34	>0.99	1.9	**0.0002**	0.011*	2.3	**<0.0001**	0.0005*
**miR-212**	30 ± 23	1.3	0.20	0.93	1	0.98	>0.99	3.0	**<0.0001**	0.004*	2.2	**0.0003**	0.019*
**miR-449a**	20 ± 12	2.0	0.0020	0.29	1.2	0.12	>0.99	2.8	**<0.0001**	0.004*	1.7	0.0015	0.058

The mean count is presented as mean normalized read counts ± S.D. across all samples (4 horses). *P* denotes raw *p*-value from the mixed linear model. *Q* denotes the *q*-value (adjusted p-value after the Benjamini-Hochberg procedure). Differences retaining significance (false discovery rate < 0.05) are denoted with an asterisk. F.C. = fold change.

**Fig 2:**
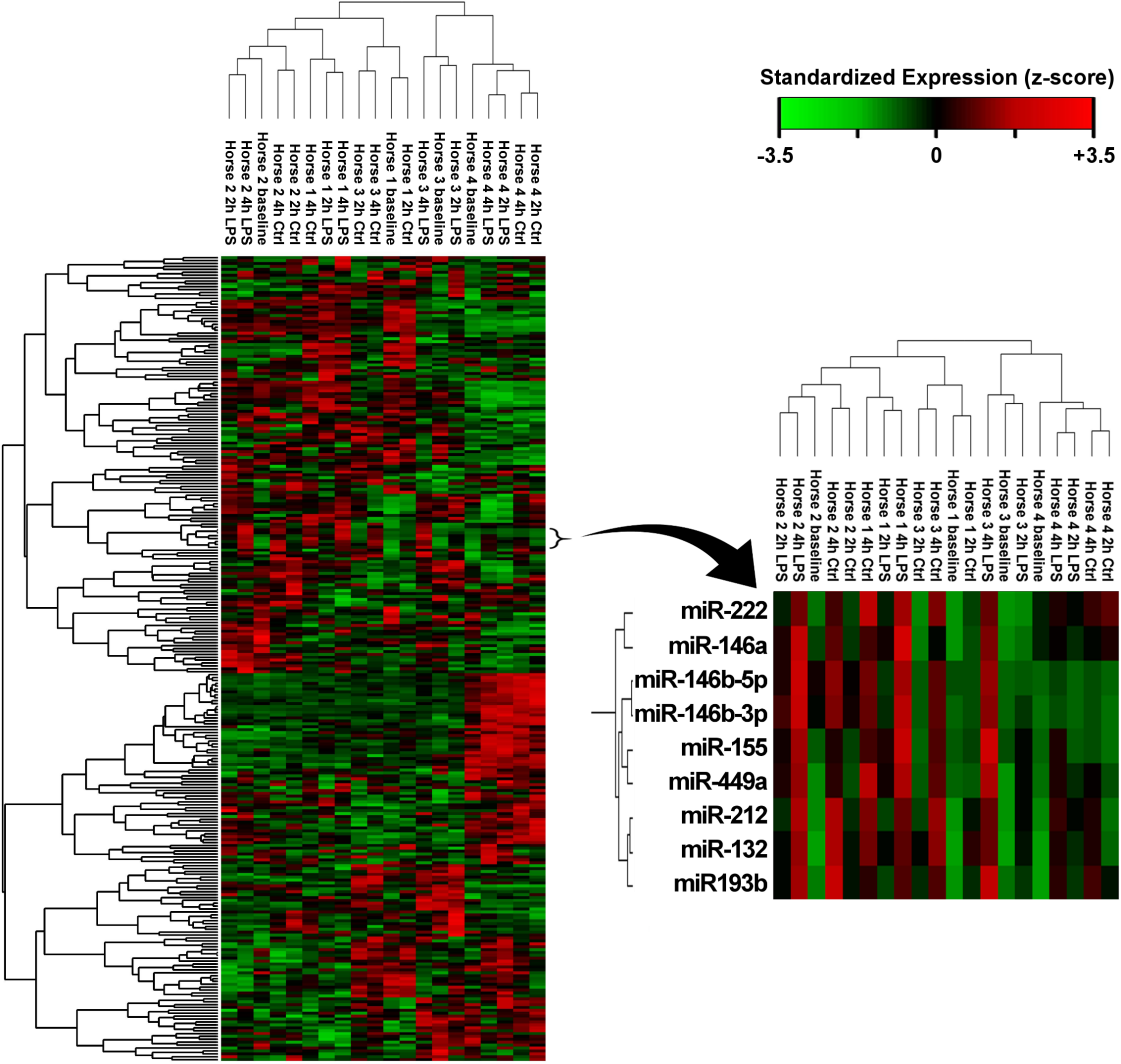
Hierarchical clustering analysis of transcriptome-wide miRNA expression patterns from NGS data. The data shown incorporates expression in baseline samples and samples cultured with or without LPS for 2 or 4 hours. Expression data have been standardized as *z*-scores for each miRNA. The subcluster inset shows 9 miRNA sequences that cluster according to expression changes with time and LPS stimulation.

Two-way hierarchical clustering analysis (Fig 2) showed that expression patterns for individual samples clustered together principally according to horse of origin rather than treatment. A subcluster of nine miRNAs was identified that segregated according to expression changes with treatment and time. This cluster (inset in Fig 2) included miR-155 and seven miRNAs with statistically significant time effects.

#### qRT-PCR validation of expression changes

Three miRNAs were selected for qRT-PCR validation of expression changes, and further investigation of changes over time, in the larger sample set. MicroRNA-155 was selected due to its significant LPS-induced expression change, whereas miR-146a and miR-146b were selected due to strong time effects, trends towards an LPS effect, high expression and known roles in TLR regulation in other species. Data were normalized to the geometric mean of two endogenous controls, miR-26a-5p and miR-103. These were selected due to high stability across groups in NGS expression as assessed by Normfinder (S2 File) and by differential expression analysis in limma, in addition to high basal expression, previously published evidence of superior stability in hematopoetic samples compared to commonly used controls such as U6 rRNA [33], and availability of validated commercial assays.

**Fig 3:**
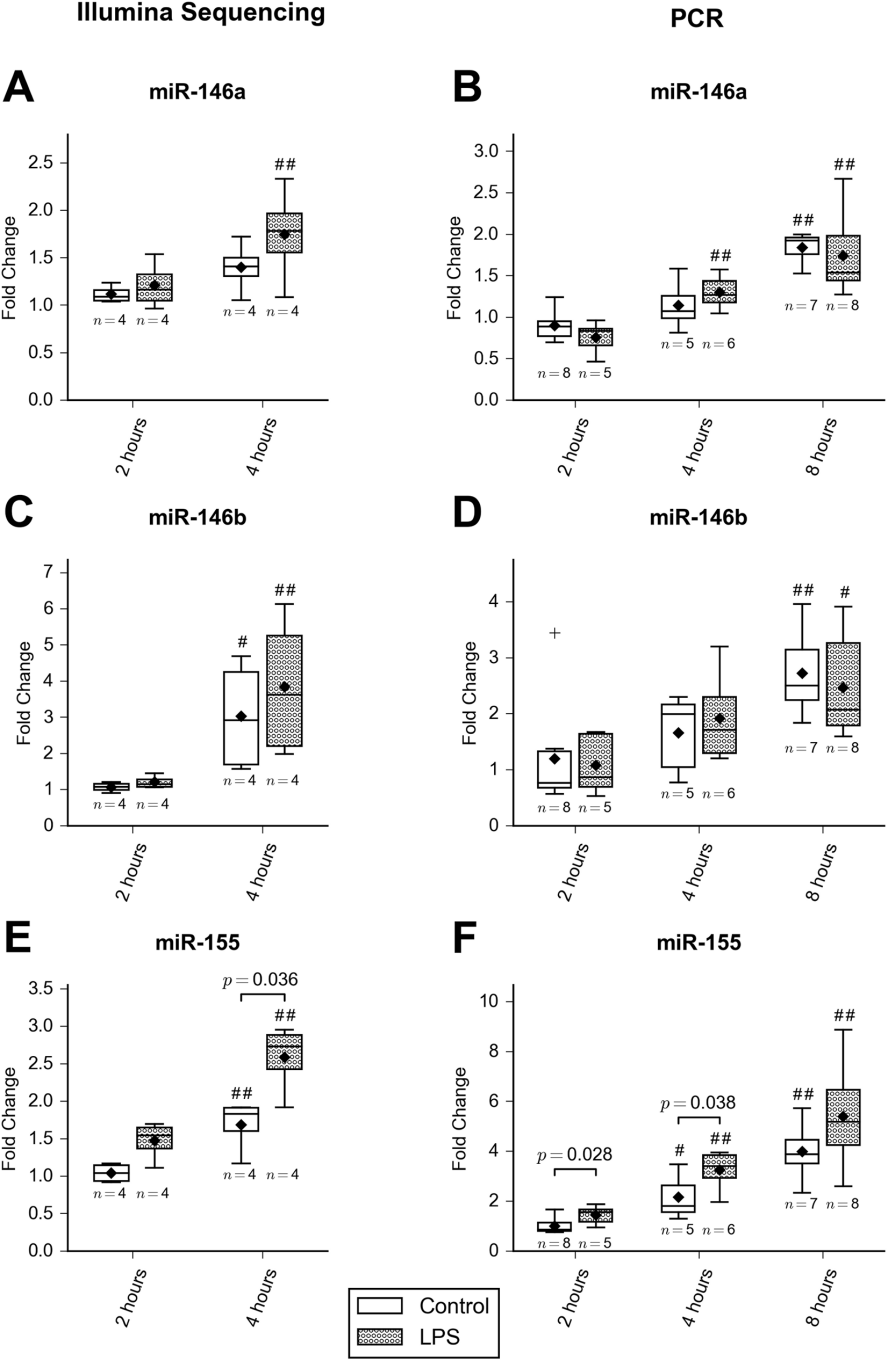
Expression changes with time in miR-146a, miR-146b and miR-155. A, C and E: Expression changes at 2 and 4 hours as determined by NGS. Expression data are presented as fold change in normalized read count from unstimulated samples at time 0. B, D and F: Expression changes from 2–8 hours as determined by qRT-PCR. Data have been normalized to the endogenous controls miR-26a and miR-103, and are expressed as fold change from unstimulated samples at time 0 (2^-ΔΔCT^). The whiskers denote twice the interquartile range, and outliers are indicated with a ‘+’ symbol. Mean is indicated with a diamond symbol, and median with a horizontal line. *P*-values for all significant differences between LPS-stimulated and control cells are given. For effects of time, significant differences from the 2-hour time point within each treatment group are indicated by # (*p* < 0.05) or ## (*p* < 0.001).

The qRT-PCR (Fig 3) analysis confirmed a significant effect of LPS on miR-155 expression (*F* = 11.9, *p* = 0.0015), with 1.4-fold upregulation at two hours (*p* = 0.028) and 1.6-fold upregulation at four hours (*p* = 0.038) in LPS-treated cells compared to controls. The LPS-induced increase at eight hours (1.3-fold) did not reach statistical significance (*p* = 0.13). No effect of LPS was evident for miR-146a (*p* = 0.57) or miR-146b (*p* = 0.97). All three miRNAs increased with time (*p* < 0.0001), but there was no significant treatment-time interaction. The magnitude and time course of expression changes for miR-155 was similar between NGS and PCR analysis (Fig 3).

### Cytokine production

**Fig 4:**
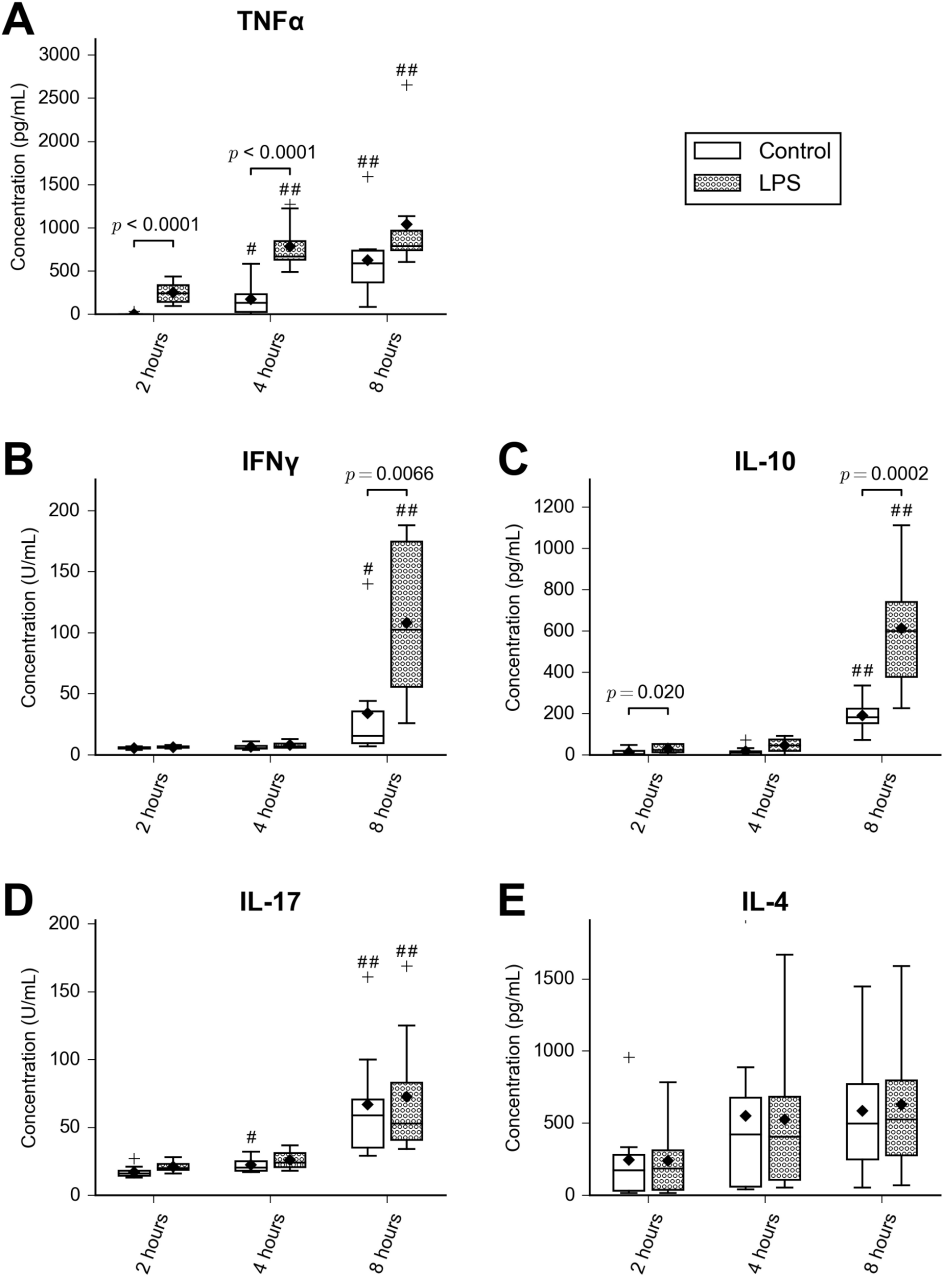
Cytokine concentrations in supernatants from cultured equine PBMCs. A) TNFα, B) IFNγ, C) IL-10, D) IL-17 and E) IL-4. *n* = 8 for all groups. The whiskers denote twice the interquartile range, and outliers are indicated with a ‘+’ symbol. Mean is indicated with a diamond symbol, and median with a horizontal line. *P*-values for all significant differences between LPS-stimulated and control cells are given. For effects of time,significant differences from the 2-hour time point within each treatment group are indicated by # (*p* < 0.05) or ## (*p* < 0.001).

Supernatant TNFα concentration increased rapidly in LPS-stimulated cells (Fig 4), with a significant increase compared to controls at two and four hours (*p* < 0.0001), falling below statistical significance at eight hours (*p* = 0.092). The increase in control cells between two and eight hours suggested some stimulation of these cells during isolation and culture. Interleukin-10 and IFNγ showed a later increase at eight hours, significantly greater in stimulated cells compared to controls (overall treatment effect *p* < 0.0001 for IL-10, *p* = 0.0035 for IFNγ). Interleukin-4 and IL-17 increased with time (*p* = 0.028 and *p* < 0.0001, respectively) but showed no effect of LPS (IL-4, *p* = 0.99; IL-17, *p* = 0.97). Interferon α was not detected in any sample.

### Associations between miRNA expression and cytokine production

**Fig 5:**
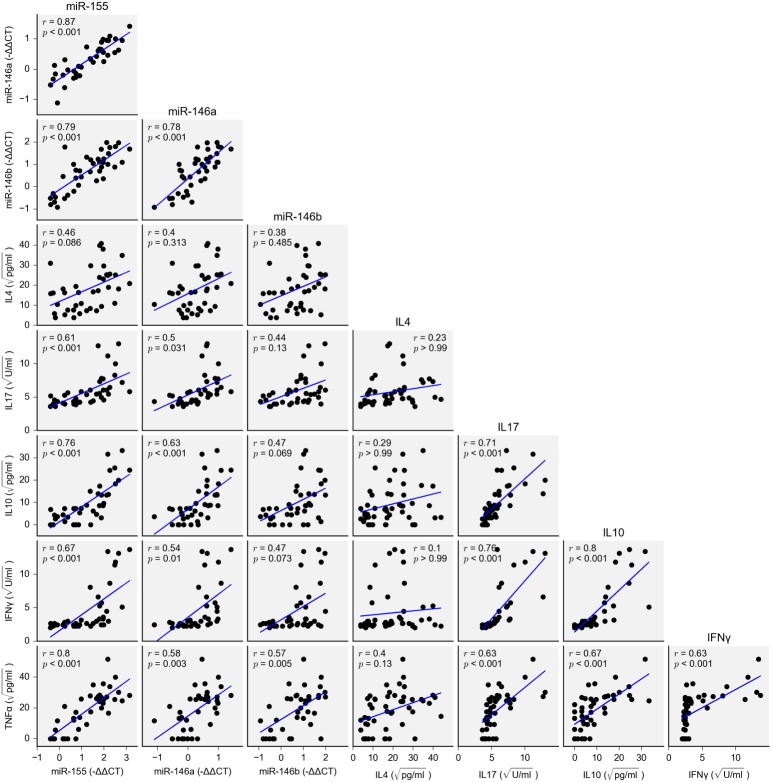
Correlation matrix of associations between miRNA expression (on qRT-PCR) and supernatant cytokine concentration. PCR data have been normalized to the endogenous controls miR-26a and miR-103. Cytokine data have been square-root transformed. The best fit line is indicated in blue. Pearson’s correlation coefficient (*r*) and Bonferroni-adjusted *p*-value are displayed for each pair.

The time course of expression change in miR-155 paralleled that observed for supernatant TNFα concentration. Pairwise correlations between the cytokines TNFα, IL-10, IFNγ, IL-4 and IL-17, and the -ΔΔCT values for the miRNAs miR-155, miR-146 and miR-146b, are shown in Fig 5. MicroRNA-155 correlated strongly with miR-146a (*r* = 0.87, *p* < 0.001) and miR-146b (*r* = 0.79, *p* < 0.001), and showed moderate to strong correlations with the cytokines TNFα, IL-10, IFNγ and IL-17 (*r* = 0.61–0.80). Statistically significant but weaker correlations (*r* = 0.50–0.63) were present between miR-146a and these cytokines, while miR-146b was correlated with TNFα (*r* = 0.57, *p* = 0.005) and the other miRNAs only. Interleukin-4, which is not typically associated with the LPS response, was not correlated with any miRNA.

### Functional analysis of equine miR-155 targets

TargetScan analysis for miR-155 yielded 262 potential targets with a total context+ score < -0.1. Three additional immune-related targets were identified using the alternative bioinformatic algorithm RNA22. Full details are given in the supporting information (S3 File).

Predicted targets for miR-155 included 34 genes related to immune responses, of which 26 were associated with innate immunity, and seven with TLR4 signaling. Notable predicted targets included suppressor of cytokine signaling-1 (SOCS1), ‘TGFβ-activated kinase 1 and MAP3K7-binding protein 2’ (TAB2), AKT serine/threonine kinase 1 (AKT1), and glucose synthase kinase 3 subunit B (GSK3B). Functional analysis of GO terms for TargetScan-predicted miR-155 targets indicated statistical over-representation of the PDGF pathway (5-fold enrichment, *p* = 0.035) and a range of biological processes (S3 File) including terms such as ‘regulation of cell differentiation’ and ‘positive regulation of signal transduction’, but no terms specifically associated with immune function.

### Conservation of the equine *MIR155* gene

**Fig 6:**
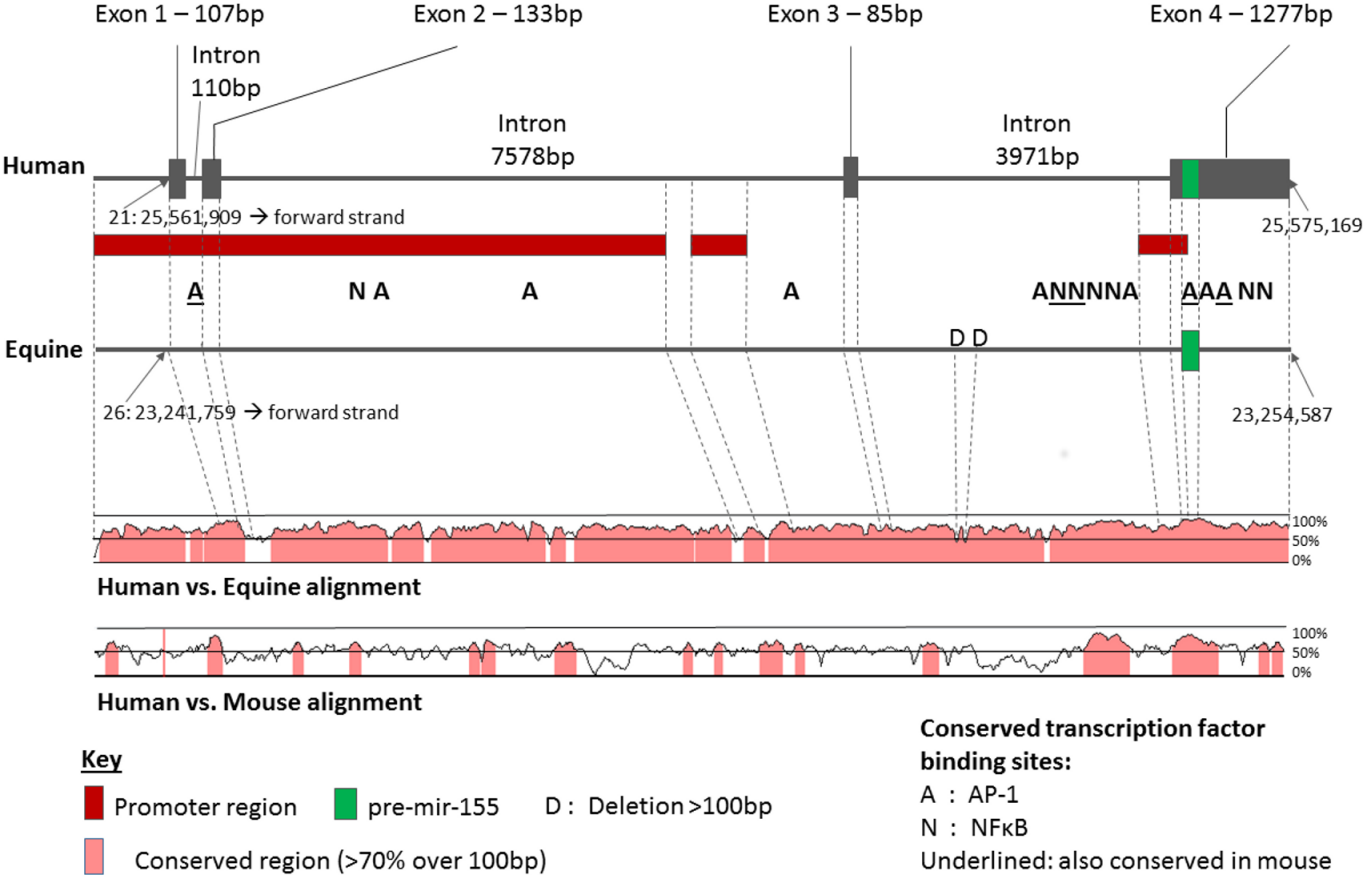
Organization and conservation of the miR-155 precursor gene. The structure of the human precursor gene is shown, with the location of the pre-miRNA-encoding region and promoter regions as described in the current assembly of the human genome. Comparison to the equine gene, and alignment with both human and murine genes is shown. Only predicted transcription factor binding sites conserved between humans and horses are indicated. Conservation is defined here as 70% nucleotide identity across a 100bp reading frame.

The equine mature miR-155 sequence is identical to the human miRNA, but differs from the murine miRNA by a single nucleotide (C versus U at position 12/23). The approximately 65-nucleotide pre-miRNA sequence differs from the human by four nucleotides, whereas the murine sequence differs by six nucleotides from the human (S1 Fig). The human miR-155 precursor gene on chromosome 21, formerly known as the B-cell Integration Cluster, contains four exons in the most recent genome assembly and Ensembl annotation. Only the 65bp sequence encoding the pre-miRNA is annotated in the current equine genome assembly (on chromosome 26). Alignment of the adjacent sequences to the human gene (Fig 6) showed a high degree of conservation of both exonic (with the exception of exon 2) and intronic regions, including promoter sequences. In contrast, alignment of the human gene with the orthologous murine gene showed lower conservation. RankVISTA analysis of conservation across all three species showed two statistically significant conserved regions, a 349bp sequence covering exon 1 and part of exon 2 (*p* < 0.0001), and a 1639bp region beginning approximately 1100bp upstream of exon 4 (*p* < 0.0001) containing the pre-miRNA. This latter highly conserved region includes a segment that, while outside the promoter region designated in the current release of the human genome, contains a number of clustered putative binding sites for NFκB and AP-1. The combination of conservation and multiple clustered transcription factor binding sites suggests that this area has promoter activity. Across the whole gene (including introns) and associated promoter regions assessed in this analysis, a total of seven putative NFκB binding sites were predicted that were conserved between humans and horses, and nine conserved AP-1 binding sites. Only two of the NFκB and three of the AP-1 binding sites were conserved across all three species.

## Discussion

In this study, we aimed to characterize the pattern of LPS-induced expression change in equine PBMCs, with a view to establishing areas of common ground between equine endotoxemia and the human response to endotoxin exposure and subsequent septic shock. Our data supported the primary hypothesis that LPS induces differential miRNA expression in equine PBMCs. Our results further identified substantial commonalities in both basal expression and in the LPS response between horses and humans, and additionally demonstrated patterns of co-expression of other relevant miRNAs that warrant further investigation.

### Characterization of the miRNA transcriptome in equine PBMCs

These data provide the first description of miRNA expression in equine leukocytes, building on previous studies in other equine tissues, and provide further experimental validation of equine miRNAs predicted *in silico*.

The equine PBMC transcriptome reported here has similarities to both the circulating plasma pool of miRNAs [16] and the miRNA transcriptome in whole blood [34] in the horse, most likely because PBMC-derived miRNAs contribute to these pools. For example, let-7g, miR-21 and miR-191a are among the most abundant miRNAs in both PBMCs and plasma of horses, while miR-150 features prominently in PBMCs and whole blood. Clear similarities with expression pattern in human PBMCs are also apparent. In a study of human patients with liver disease and healthy controls, miR-21 was the most abundant in PBMCs from all donors, while the second most abundant in eight of twelve samples was either let-7g or miR-26a (ranked second and third in horses) [35]. Other highly expressed miRNAs common to human and equine PBMCs include miR-191a and miR-150 [36, 37]. It is however notable that there has been marked variation in the reported human PBMC transcriptome between studies, suggesting an influence of subject selection, cell processing, or measurement methodology.

Many equine miRNAs previously considered specific to colon, muscle or liver, based on initial sequencing of these tissues [15], were also detected in PBMCs in this study. These included some of the most abundant miRNAs in this population of leukocytes, including let-7g and miR-148a (considered liver-specific), and miR-150 and miR-142 (considered colon-specific). Furthermore, miRNAs of likely functional significance in PBMCs, including miR-146a, miR-146b and miR-155 were previously designated as colon-specific. The presence of macrophages and lymphoid tissue in the colon could account for some of this overlap. miRNA profiles have not been established in many equine tissues, and as reporting is often incomplete even for those tissues with available data, it cannot be determined whether any of the miRNAs detected in the present study are truly specific for PBMCs.

### miR-155 is the principal LPS-induced miRNA in equine PBMCs

These data on LPS-induced changes in miRNA expression in complement recently published data on global gene expression changes in response to LPS in equine PBMCs [38]. This is one of very few studies published to date to use NGS to investigate miRNA responses to LPS in any species [39, 40]. Next Generation Sequencing has considerable advantages over microarray platforms in terms of dynamic range and sensitivity, and can discriminate between miRNAs that differ by a single nucleotide [41]. While qRT-PCR is often considered the ‘gold standard’ for validation of expression changes, a direct comparison of platforms for expression profiling has suggested that Illumina sequencing has a comparable specificity (71%) but slightly higher sensitivity than PCR (87% versus 83%) for correct identification of differentially expressed miRNAs. Although overall correlation between the two methods is good, discrepancies in observed expression change are common for individual sequences [42]. There is therefore no widespread consensus as to the optimal technology for differential expression analysis.

The expression change observed in this study supported the primary hypothesis that LPS induces differential miRNA expression, although the expression changes observed were not as extensive as anticipated. The LPS-induced upregulation of miR-155 in this model is consistent with the majority of human and murine models and cell lines studied to date [7, 43–46]. Contrary to predictions, no effect of LPS on expression of miR-146a or miR-146b in equine PBMCs could be demonstrated in the current model, although the trends observed with time suggest that these could warrant further investigation. Lipopolysaccharide-induced upregulation of miR-146a has been observed in numerous models, especially in pure cultures of cells of the monocyte-macrophage lineage [4, 44, 45, 47]_._ Lack of an LPS-induced miR-146a response is not unprecedented, however, and other studies have reported no change in expression in human B cells [4], or in CD4+, CD8+ and CD14+ cells derived from human cord blood or adult peripheral blood [46].

The limited extent of LPS-induced expression changes in this model was surprising. Limited statistical power due to small sample size in the NGS analysis could certainly have contributed. Given the likelihood of type II errors, miRNAs with LPS effects that did not retain statistical significance, or which showed evidence of co-expression with miR-155, should be considered for future investigation. Time-dependent expression changes, without an obvious LPS effect, for miRNAs such as miR-146a could have been a result of non-specific background stimulation of the cells, potentially masking minor treatment effects. This could have arisen from the isolation and culture procedures themselves, from the presence of other TLR4 ligands such as host-derived heat-shock proteins [48], or from small quantities of contaminating LPS, which is ubiquitous in the horse’s environment. Spontaneous production of the cytokines studied here, of a comparable magnitude to the time-induced changes observed, have been previously reported in similar culture systems with equine PBMCs and are therefore not unexpected [49]. The differences in production of key LPS-induced cytokines and in miR-155 expression do however indicate that sufficient differential stimulation was achieved to observe an LPS effect.

Lipopolysaccharide dose, duration of stimulation and cell population can all influence the induced expression profile. Delayed miRNA responses to LPS, such as the IL-10-mediated induction of miR-146b described after eight hours in human monocytes [45], may not have been detected in the present model, but changes in many LPS-responsive miRNAs can be observed within two hours [7, 45, 50]. The use of a single LPS concentration may represent a limitation of this study, as supra-physiological or suboptimal doses could produce different miRNA expression patterns. Higher LPS doses have been used in many studies of miRNA responses in other models, but given the horse’s exquisite sensitivity to LPS these doses may not be appropriate or clinically relevant in an equine model. Previous studies have shown maximal cytokine expression in equine monocytes with LPS concentrations below 1 ng/mL, and clinical cases rarely exceed this level [51–53]. Reconfirming the findings of this study with lower clinically relevant doses would however be worthwhile, as ultra-low LPS concentrations in experimental models can induce responses that differ markedly from more commonly used doses [54].

The mixed PBMC population used here is in some ways more physiologic than a pure cell line, as leukocytes do not function in isolation, and there is extensive communication between lymphocytes and monocytes / macrophages to direct the immune response. For example, the increase in IFNγ in LPS-stimulated cells in this model, probably in part from T-lymphocytes [55], could have blunted the effects of LPS on miR-146a, miR-146b and miR-132, as observed in human cord blood CD14+ cells [46]. Expression changes in a single cell type may be masked or diminished in apparent magnitude if other cells in the sample respond in a different manner. It is thus possible that analysis of the pooled population missed biologically significant expression changes in specific subsets of equine PBMCs. Involvement of miRNAs in the regulation of the inflammatory response to LPS could therefore be more extensive than suggested by these results.

### Regulation of miRNA expression in LPS-stimulated cells

MicroRNA-155 is one of the most studied of all miRNAs, and is implicated in a number of disease processes. The mechanisms of miR-155 induction vary according to cell line and stimulus, but the transcription factors NFκB and AP-1 both play prominent roles in the LPS response [56, 57]. An AP-1 binding site just upstream of exon 2 (Fig 6) is thought to play a key role, and it is therefore notable that this site is conserved in the horse. Multiple conserved NFκB binding sites were also detected in the equine genome, and thus it is likely that regulation of expression via this pathway is similar in horses and humans. This is further supported by the temporal association between miR-155 expression changes and production of TNFα, which is induced by NFκB [58].

A number of miRNAs clustered together with miR-155 on the hierarchical clustering analysis (Fig 2), including miRNAs such as miR-146a, miR-146b and miR-132 that had significant increases in expression with time. Interestingly, the majority of these miRNAs have associations with LPS and toll-like receptor signaling in other species. Clustering on this analysis is suggestive of co-regulation [27]. This could be related to LPS, stimulation of TLRs by other ligands, or to some other as yet unidentified stimulus. This close relationship suggests that, while our data provide no evidence of direct LPS induction, these miRNAs should be considered for further study into their potential roles in innate immunity.

### Functional significance of miR-155 upregulation

**Fig 7:**
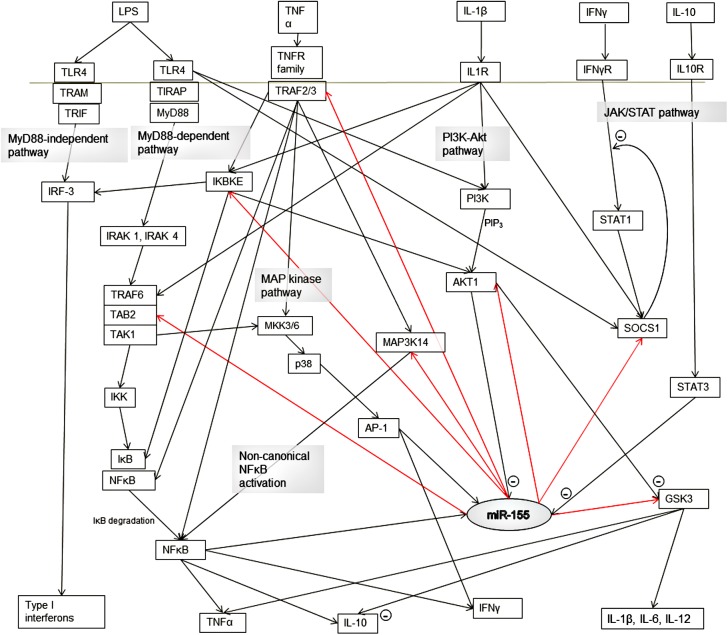
Interactions between miR-155, its targets, cytokines and transcription factors in innate immune pathways. Pathways have been simplified. Targets shown here (indicated by red arrows) are predicted in the horse and have supportive experimental evidence in other species.

Although statistical enrichment of immune pathways among predicted targets was not demonstrated, bioinformatic predictions indicated a number of possible targets for miR-155 in the toll-like receptor signaling cascade, as well as other aspects of the innate and adaptive immune system (Fig 7). These included SOCS1, TAB2, AKT1 and GSK3B.

Suppressor of cytokine signaling-1 is one of the best studied targets of miR-155. Its anti-inflammatory effects include inhibition of the JAK/STAT signaling pathway (which mediates the effects of IFNγ and other cytokines), and direct effects on TLR signaling [59]. By suppressing LPS-induced production of SOCS1 (an inherent negative feedback system), upregulation of miR-155 would be expected to augment the effects of inflammatory cytokines in endotoxemia. There is strong evidence that miR-155-mediated suppression of SOCS1 has biological significance in infection and autoimmunity [60]. This interaction has for example been implicated in the reduction in LPS-induced liver injury and reduced TNFα production observed with a miR-155 inhibitor in a mouse model [61]. In other contexts, however, the effect of miR-155 on SOCS1 may in fact be protective. For example, miR-155 knockout renders mice more susceptible to infection with *Mycobacterium tuberculosis* [62].

Targeting of other genes by miR-155 is predicted to have anti-inflammatory effects. The signal transduction protein TAB2 is a key element in the MyD88-dependent TLR and IL-1 pathways, and can activate both the NFκB pathway and the MAPK pathway. Suppression of this protein is therefore expected to reduce the inflammatory response to LPS and IL-1 [56, 63, 64]. A number of targets were also predicted in the PI3 kinase / AKT signaling pathway, including PI3K regulatory subunit alpha, AKT1 and GSK3B. This pathway regulates diverse cellular processes including apoptosis, metabolism and inflammatory processes, and can be activated by many receptors including toll-like receptors. Based on their respective roles in the pathway (Fig 7), targeting of PI3K and AKT1 would be expected to augment the production of pro-inflammatory cytokines such as TNFα, IL-1ß, IL-6 and IL-12, while suppressing anti-inflammatory cytokines such as IL-10, promoting a T_h_1-type T-cell mediated immune response [65]. Suppression of GSK3 would have the opposite effect. As AKT1 also suppresses miR-155 production, suppression of AKT1 by miR-155 would form a positive feedback loop, enhancing its own expression. The predominant effect of the LPS-induced upregulation of miR-155 on this pathway is therefore difficult to predict, and will depend on the relative efficiency of repression of each target. *In silico* target predictions are not always reliable, and so manipulation using miRNA mimics or inhibitors will be necessary to confirm the effects of this miRNA on the above targets and inflammatory responses in the horse.

Overall, data in other species indicate that the significant upregulation of miR-155 in this model is likely to have predominantly pro-inflammatory effects. For example, ectopic expression of miR-155 reverses the effect of dexamethasone on TNFα, IL-6 and nitric oxide production in LPS-stimulated macrophages [66]. In clinical disease, it is often the resultant systemic inflammatory response rather than the presence of LPS *per se* which causes much of the morbidity and mortality, particularly in equine conditions such as gastrointestinal disease without substantial bacteremia. In such cases, miR-155 could exacerbate the clinical syndrome.

### The equine miRNA response to LPS as a model for human disease

Substantial parallels exist between equine endotoxemia and septic shock in human patients. Horses and humans are both exquisitely sensitive to the effects of LPS. The maximum tolerated intravenous infusion dose in human volunteers is around 4 ng/kg, and the lethal dose is estimated to be 1–2 μg [11]. The LD_50_ in mice, in contrast, has been reported to be between 1.6 and 25.6 mg/kg [12]. Horses have an intermediate sensitivity. Intravenous infusion of 20 ng/kg induces a transient systemic inflammatory response [9], and while the LD50 is unknown, intravenous doses of 50–200 μg/kg are lethal [67]. The closer similarity in LPS sensitivity between horses and humans could make equine endotoxemia attractive as an alternative model for human Gram-negative sepsis. Although some aspects of the disease phenotype are different (for example the prominence of hoof-lamellar interface pathology in horses), other facets of the disease such as cardiopulmonary shock and acute lung injury are similar [68].

The miRNA response to LPS in human inflammatory cells has been investigated in a number of models, usually by microarray or qRT-PCR. Except for increases in miR-155, there has been remarkably little consensus between studies, likely due in part to differences in the cell populations studies and to methodological differences. Other common, but not universal, expression changes have included upregulation of miR-146a and/or miR-146b in most models (but not in B-cells or PBMCs), upregulation of miR-9, miR-18a and miR-132, and downregulation of miR-125b [4, 7, 44–46, 63]. The whole blood response to low-dose intravenous LPS in healthy volunteers has also been studied, and the limited range of differentially-expressed miRNAs (including downregulated miR-150) did not overlap with those changes seen in specific cell populations in vitro [69].

The finding of this study that miR-155 is the principal LPS-induced miRNA in horses, as in other species, supports the hypothesis that the miRNA response is comparable with humans and lends some support to the use of an equine model to study this aspect of the response to endotoxin. Moreover, the miR-155 precursor gene and adjacent regulatory elements examined here have a much closer sequence identity to humans than do the mouse equivalents. Some differences in expression patterns between the species however indicate the need for caution in extrapolating from horses to humans. Lack of upregulation of miR-146a and miR-146b could represent a weakness of an equine model such as this, although this is potentially consistent with some human models using patient-sourced cells [46] as opposed to cell lines. Nonetheless, valuable insight can be gained from the comparative study of naturally-occurring disease between species, and the equine model offers a unique addition to the tool box of options for exploration of events that orchestrate the inflammatory cascade directing life-threatening endotoxemia and septic shock [70].

### Conclusions

This study represents the first investigation into the involvement of miRNAs in equine innate immunity. Consistent with the primary hypothesis, differential miRNA expression in response to LPS was demonstrated in equine PBMCs, although only for a single miRNA, miR-155. *In silico* functional analysis suggested that this is likely to have a similar function in the horse as in other species. Both this miRNA and other miRNAs without clear LPS-induced expression changes, such as miR-146a, are likely to influence the phenotype and magnitude of the systemic inflammatory response to LPS in the horse. They could thus provide potential therapeutic targets for modulation of local or systemic inflammation. The expression patterns documented by this *in vitro* model provide a starting point for such research.

## Supporting information

S1 Fig Neighbor joining tree to show similarities of human pre-miR-155 to 18 other species. (PDF)

S1 File Details of samples used for Next Generation Sequencing, PCR and cytokine analysis. Details are provided of the time points, treatments, sample numbers and horse demographics for each stage of the analysis.
(XLSX)

S2 File Details of miRNA expression in equine PBMCs. A: Mature miRNAs, including sequences, loci on equine chromosomes and expression data. B: Screening of candidate endogenous controls for expression stability using Normfinder.
(XLSX)

S3 File Equine target predictions for miR-155. A: miR-155 target predictions generated by TargetScan. B: Target predictions for eca-miR-155 generated using RNA22, from a list of 51 candidate genes with experimental evidence in other species. C: Statistical over-representation analysis of Gene Ontology biological function terms for TargetScan miR-155 target predictions.
(XLSX)
