# Supplementary material for: Characterization of basal and lipopolysaccharide-induced microRNA expression in equine peripheral blood mononuclear cells using Next-Generation Sequencing
Source: PLoS One. 2017 May 26;12(5):e0177664. doi: 10.1371/journal.pone.0177664 (PMC5446123; doi:10.1371/journal.pone.0177664)

Supporting Figure S3. Neighbor joining tree to show similarities of human pre-miR-155 to 18 other species.

'Homo sapiens' forms the root of the tree. The tree was constructed using the NCBI Blast tree viewer (https://blast.ncbi.nlm.nih.gov/Blast.cgi) based on BLASTN analysis of the annotated miR-155 precursor sequence for each species.

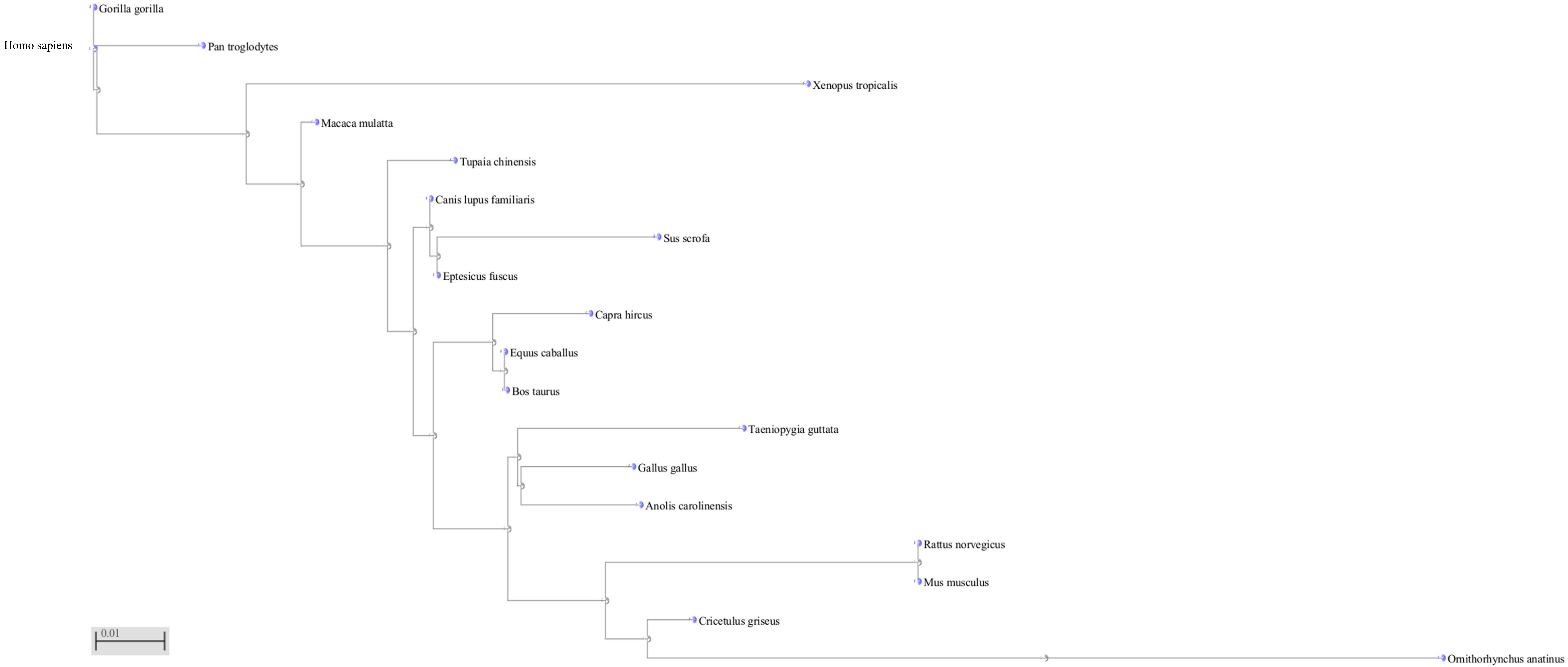

Supplement: S1 Fig — (PDF) [file pone.0177664.s001.pdf]
